# A pilot dose finding study of pioglitazone in autistic children

**DOI:** 10.1186/s13229-018-0241-5

**Published:** 2018-11-26

**Authors:** Lucia Capano, Annie Dupuis, Jessica Brian, Deepali Mankad, Lisa Genore, Rianne Hastie Adams, Sharon Smile, Toni Lui, Dina Odrobina, Jane A. Foster, Evdokia Anagnostou

**Affiliations:** 1 Holland Bloorview Kids Rehabilitation Hospital, University of Toronto, 150 Kilgour Road, Toronto, ON M4G 1R8 Canada; 2 Sick Kids, University of Toronto, 555 University Avenue, Toronto, ON M5G 1X8 Canada; 3 Holland Bloorview Kids Rehabilitation Hospital, 150 Kilgour Road, Toronto, ON M4G 1R8 Canada; 4 St. Joseph’s Health Care, McMaster University, 50 Charlton Avenue East, Hamilton, ON L8N 4A6 Canada

**Keywords:** Pioglitazone, Autism spectrum disorder, Cytokines, Treatment, Clinical trial, Efficacy, Safety profile, Inflammation, Drug therapy, Physiological effects of drugs, Maximum tolerated dose (MTD)

## Abstract

**Background:**

Pioglitazone is a promising compound for treatment of core autism spectrum disorder (ASD) symptoms as it targets multiple relevant pathways, including immune system alterations.

**Objective:**

This pilot study aimed to elucidate the maximum tolerated dose, safety, preliminary evidence of efficacy, and appropriate outcome measures in autistic children ages 5–12 years old.

**Methods:**

We conducted a 16-week prospective cohort, single blind, single arm, 2-week placebo run-in, dose-finding study of pioglitazone. Twenty-five participants completed treatment. A modified dose finding method was used to determine safety and dose response among three dose levels: 0.25 mg/kg, 0.5 mg/kg, and 0.75 mg/kg once daily.

**Results:**

**Maximum tolerated dose:** there were no serious adverse events (SAEs) and as such the maximum tolerated dose within the range tested was 0.75 mg/Kg once daily.

**Safety:** overall, pioglitazone was well tolerated. Two participants discontinued intervention due to perceived non-efficacy and one due to the inability to tolerate interim blood work. Three participants experienced mild neutropenia.

**Early evidence of efficacy:** statistically significant improvement was observed in social withdrawal, repetitive behaviors, and externalizing behaviors as measured by the Aberrant Behavior Checklist (ABC), Child Yale-Brown Obsessive Compulsive Scale (CY-BOCS), and Repetitive Behavior Scale–Revised (RBS-R). Forty-six percent of those enrolled were deemed to be global responders.

**Conclusions and relevance:**

Pioglitazone is well-tolerated and shows a potential signal in measures of social withdrawal, repetitive, and externalizing behaviors. Randomized controlled trials using the confirmed dose are warranted.

**Trial registration:**

ClinicalTrials.gov, NCT01205282. Registration date: September 20, 2010.

**Electronic supplementary material:**

The online version of this article (10.1186/s13229-018-0241-5) contains supplementary material, which is available to authorized users.

## Introduction

Autism spectrum disorder (ASD) is a complex neurodevelopmental condition with life-long impact on patients, affecting almost 1 in 59 children [1, 2]. The core symptoms of ASD involve varying degrees of difficulty in social communication, repetitive behavior, and restricted interests [1]. Autistic individuals^1^ also experience frequent physical and mental health comorbidity. Risperidone and aripiprazole are the only medications approved for use in ASD for the treatment of irritability and aggression. There are no pharmaceutical treatment options for the core symptoms of ASD, and perhaps more importantly, none that impact the developmental trajectory of autistic children. Part of the challenge in the development of new treatments is identifying the biological basis of the pathophysiology underlying the clinical heterogeneity in ASD.

The pathophysiology underlying ASD is under investigation. The disorder is known to have strong genetic influences and some environmental risk factors. Clinical heterogeneity in autism makes it difficult to match individuals with effective treatments. Literature evidence supports a role for immune signaling and inflammation in ASD [3–10] but current approaches have not examined or identified individual differences in biology that underlie the striking clinical heterogeneity. Of note, whether immune differences are primary to the pathophysiology or a later epiphenomenon has not been clarified. In an effort to advance our understanding of the role of the immune system in ASD, we assessed baseline and treatment-related changes in peripheral cytokines in an exploratory fashion, as part of a maximum tolerated dose (MTD) study with pioglitazone (NCT01205282).

## Evidence of abnormal central nervous system immune signaling and inflammation in ASD

Evidence of immune system alterations within the brains of autistic individuals comes from examination of post-mortem brain tissue, cerebrospinal fluid (CSF), and positron emission tomography (PET) studies. Post-mortem tissue analysis has demonstrated activated microglia and astrocytes in several areas of cerebral cortex, cingulate gyrus, white matter, and cerebellum [11–13]. Recently, elevated microglial activation was demonstrated in vivo using PET [14]. The ages of individuals in these studies varies from early childhood to adulthood suggesting that such neuroinflammation may affect the presentation of autism across the life span. In comparison to controls, autistic individuals have elevated levels of glial fibrillary acidic protein (GFAP), confirming astroglial activation (reviewed in [15]). Increased expression and release of cytokines and chemokines, the mediators of inflammatory reactions in the CNS, have also been observed. Elevated levels of proinflammatory chemokines reported in ASD include macrophage chemoattractant protein-1 (MCP-1), thymus activation-regulated chemokine (TARC), proinflammatory cytokines interleukin-6 (IL-6), interleukin-10 (IL-10), macrophage chemoattractant protein-3 (MCP-3), eotaxin, eotaxin 2, macrophage derived chemokine (MDC), chemokine-β8 (Ckβ8.1), neutrophil activating peptide-2 (NAP-2), monokine induced by interferon-ϒ (MIG), and B-lymphocyte chemoattractant (BLC) [11]. Studies comparing CSF in autistic children versus controls have shown elevated pro-inflammatory cytokines tumor necrosis factor-α (TNF-α), IL-6 and granulocyte-macrophage colony stimulating factor (GM-CSF), Th1 cytokine (IFN-ϒ), and chemokine (IL-8) (reviewed in [15]). Other studies have found that patients with ASD produce lower levels of anti-inflammatory cytokines IL-10 and TGF-1β [16, 17].

## Evidence of abnormal peripheral immune system signaling and inflammation in ASD

Studies comparing cytokine levels from the peripheral immune system (from blood samples) to CNS (via CSF) do not find a clear relationship, suggesting that blood samples may not provide a clear indication of CNS immune activity [4]. However, cross-talk between peripheral cytokines and the CNS are known to impact behavior [18, 19]. Elevated levels of pro-inflammatory cytokines and reduced levels of anti-inflammatory cytokines have been observed in autistic children compared to controls [20–22]. A recent meta-analysis of studies of peripheral cytokine levels in ASD compared to controls demonstrated higher concentrations of interleukin (IL)-1β, IL-6, IL-8, interferon-gamma, eotaxin, and MCP-1, while concentrations of transforming growth factor-β1 (TGF-1β—anti-inflammatory cytokine) were significantly lower [20, 21]. In a study of 65 autistic children compared to healthy controls, level of IL-8 in the blood was found to be significantly higher, while IL-10 was significantly lower [23]. However, in a longitudinal study of 104 autistic children compared to controls, there was no difference in levels of traditional markers of active inflammation such as IL-6, TNF-α, and IL-1β [4].

Evidence is accumulating that demonstrates a link between elevated cytokine levels and behavior. Ashwood et al. [24] found that increased IL-6 level in autistic children was associated with increased stereotypy scores on the Aberrant Behavior Checklist (ABC). Tsilioni et al. [25] observed that both IL-6 and TNF-α were decreased significantly in autistic children when they took luteolin a natural flavonoid, and children with high levels of both IL-6 and TNF-α at baseline had significant improvement of all subscales of the Vineland adaptive behavior scale following this treatment. Reduced TGF-1β was seen in ASD versus controls and correlated with lower adaptive behavior and worse behavioral symptoms [16]. Similarly, another study found that levels of TGF-β1 and IL-23 were significantly decreased in the plasma of autistic children in comparison to healthy controls with a negative correlation between TGF-β1, IL-23, and IL-17 with the severity of autism [26]. Additionally, IL-17A was found to be significantly higher in the serum of autistic children than healthy controls, and higher levels correlated with severity of autism [7].

## Pioglitazone pharmacodynamics and studies in ASD

Overall, the studies discussed above demonstrate that alterations in both peripheral and CNS cytokines are observed in ASD and identifying drug treatments that normalize some of these immune markers may be a fruitful avenue of research. Pioglitazone is an agonist of peroxisome proliferator activated receptor (PPAR)-ϒ, and is part of a drug class called thiazolidinediones (TZDs). PPAR-ϒ is a nuclear hormone receptor which modulates insulin sensitivity but is also shown to have anti-inflammatory effects [27]. The anti-inflammatory functions of PPAR-ϒ have received much attention since its agonists have been shown to exert a broad spectrum of protective effects in several animal models of neurologic and cardiovascular diseases [28, 29]. To date, pioglitazone has been shown in vitro and in early in vivo studies to have a variety of effects of interest in the pathophysiology of autism. Those include inhibition of expression of inflammatory cytokines (IL-1b, IL-6, and IL-8) [29], and reduction in TNF-α [30], but also affects on mitochondrial function and NMDA glutamate receptor activity [31, 32], which may also be important in ASD pathophysiology [33–35].

Studies of pioglitazone in autistic individuals include one case series and a pilot double-blind placebo-controlled study of pioglitazone as adjunctive treatment to risperidone [36, 37]. The authors of the case series reported on 25 children (mean age 7.9 ± 0.7 years) with autism who were treated with 30–60 mg of pioglitazone a day, over 3–4 months. They reported improvements in four of five scales of the ABC and no side effects other than mild elevation in liver enzymes, a well-known phenomenon with this medication. A drawback of this study was the choice of pioglitazone dose. The FDA-approved maximum adult dose is 45 mg. Lower doses have been used in the trials of type II diabetes and studies for use of pioglitazone in central nervous system diseases such as multiple sclerosis. Additionally, only the ABC was piloted as an outcome measure. Other outcome measures may prove to be more sensitive. Ghaleiha et al. [37] randomized autistic children receiving risperidone to pioglitazone or placebo adjunct treatment. Forty-four patients aged 4–12 years with a score of more than 12 on the Aberrant Behavior Checklist-Community (ABC-C) were included in a 10-week study. They found a significant reduction in irritability, lethargy/social withdrawal, and hyperactivity/non-compliance, but not for stereotypic behavior and inappropriate speech subscales in the pioglitazone group compared with the placebo group. Vomiting and headache were the most frequent reported side effects. Of the two studies conducted on pioglitazone in ASD to date, one used doses above the recommended maximum for adults, while the other used pioglitazone as adjunct therapy. Therefore, both the safety and efficacy of pioglitazone in ASD requires further study.

In this study, we conducted a weight-based evaluation of maximum tolerated dose using a modified dose finding method, where maximum dose was based on the FDA and Health Canada approved adult maximum. We used several outcome measures sensitive to changes in behavior, to help guide the design of a larger multisite placebo-controlled trial. In an exploratory fashion, we measured levels of cytokines (IL-1β, IL-6, TNF-α, and IL-10) pre and post treatment as potential predictors of response to drug. The design was a phase II, 16-week, single-blind, single arm, placebo run-in, modified MTD study of pioglitazone in autistic children.

## Methods

### Participants

Eligible participants were 5–12 years old with a Diagnostic and Statistical Manual of Mental Disorders 4th edition, text revision (DSM-IV-TR [38]) diagnosis of autistic disorder or Asperger syndrome (autism spectrum disorder) supported by the Autism Diagnostic Observational Schedule, First or Second Edition (ADOS [39] or ADOS-2 [40, 41]) and the Autism Diagnostic Interview-Revised (ADI-R [42]). The Diagnostic and Statistical Manual of Mental Disorders 5th edition (DSM-5) was published after participant enrollment had begun; therefore, the protocol was not revised for use of DSM-5 for diagnosis. Eligibility for inclusion also required a Clinical Global Impression-Severity (CGI-S [43]) score ≥ 4 to ensure participants were experiencing functional impairment at least moderate in severity that could benefit from medication treatment. The CGI-S is a standardized measure commonly used in mental health research, for ranking illness severity (therefore for autism this would include both core and associated symptoms). All participants had normal physical examination and laboratory test results at screening visit. Participants receiving non-pharmacologic educational, behavioral, or dietary interventions at study entry were required to be stable on such interventions (continuous participation during the preceding 3 months) and to not electively initiate new or modify ongoing interventions for the duration of the study. Participants were excluded if they had a primary psychiatric diagnosis other than ASD, a current neurological disease including a movement disorder, tuberous sclerosis, fragile X or any other known genetic syndrome, or any medical condition that might interfere with the conduct of the study, confound interpretation of the study results, or endanger a participant’s well-being. Hence, patients with evidence or history of malignancy or any significant hematological, endocrine, cardiovascular (including any rhythm disorder), respiratory, renal, hepatic, or gastrointestinal disease were excluded. However, patients with stable epilepsy (defined as no seizures for 6 months) and on stable doses of antiepileptic medications (no changes in the 3 months preceding screening) were allowed to participate. Prior studies of pioglitazone have shown that individuals taking this medication had a slightly higher incidence of bladder cancer and the risk seemed to be higher in smokers. Therefore, participants were not allowed to participate if they had a family history of bladder cancer, a caregiver who smoked, or a personal history of bladder infection. Participants taking any psychoactive medication or insulin were excluded as were any female participants who were sexually active, taking the birth control pill for any reason, or pregnant. Participants were excluded if unable to tolerate venipuncture procedures for blood sampling.

The informed consent process was conducted in accordance with the Tri Council Policy Statement, the Declaration of Helsinki, and the International Council for Harmonization of Technical Requirements for Pharmaceuticals for Human Use (ICH)–Good Clinical Practice (GCP) guidelines and institutional policy. This study was approved by the Holland Bloorview Research Ethics Board.

### Study design

This phase II pilot study was designed as a 16-week, modified maximum tolerated dose study of pioglitazone in autistic children conducted at Holland Bloorview Kids Rehabilitation Hospital in Toronto, Canada. Participants were enrolled consecutively over 2 years. Our national pharmaceutical regulatory body, Health Canada, required that a maximum tolerated dose (MTD) study be conducted prior to additional clinical trials of pioglitazone for autistic children. Such studies are normally open label; however, in an effort to improve our confidence in evaluating preliminary efficacy and safety, we employed a 2-week placebo run-in and single-blind design. Parents were blinded to the timing of the 2-week placebo period. The consent process disclosed that at some point during the study, participants would get placebo, but not when. The study involved 2 weeks of placebo run-in and 14 weeks of active treatment. The placebo was identical in composition to the active drug except for the active ingredient. Compliance with the study dosing schedule was monitored with a medication diary that was provided to parents/guardians that they completed daily. Parents were the primary efficacy raters and were blinded to treatment as described above. Clinicians were not blinded, and were solely responsible for safety assessments and outcome ratings (using CGI-S, Clinician Global Impression scale-Improvement (CGI-I) [43] and the Child Yale-Brown Obsessive Compulsive Scale (CY-BOCS; [44], modified by [45]).

Participants were required to attend approximately seven study visits—screening, baseline, 2, 4, 8, 12, and 16 weeks from baseline. A modified dose finding method was used to determine safety and dose response among three dose levels (0.25 mg/kg once daily, 0.5 mg/kg once daily, and 0.75 mg/kg once daily). The dose was based on the FDA-approved maximum adult dose of 45 mg. Taking 60 kg as an average adult weight, the per kilogram maximum dose is 0.75 mg/kg. Standard toxicity protocols are based on serious adverse events (SAEs) to determine the MTD. We defined toxicity as the presence of SAEs or severe side effects deemed by the treating clinician and investigators to be likely or probably related to study drug. The most recent monograph of pioglitazone lists heart failure, hepatic insufficiency, and allergic reactions as significant side effects of pioglitazone. Five participants were studied at the first dosing level (0.25 mg/kg), and five were studied at the second dosing level (0.5 mg/kg). No serious adverse events presented at 0.25 or 0.5 mg/kg, therefore the remaining 18 participants were studied at the final dosing level of 0.75 mg/kg.

### Assessments

#### Screening/diagnostic assessments

At the screening visit, confirmation of DSM-IV diagnosis of autism or Asperger syndrome was obtained by medical history, either ADOS or ADOS-2 and ADI-R. Additionally, a comprehensive medical history and physical exam as well as a Safety Monitoring Uniform Report Form (SMURF) and Clinician’s Global Impression (CGI)-severity (Global & Social [43]) were completed by a study physician. An estimate of intellectual ability was obtained using the Stanford Binet 5th Edition. The Stanford Binet 5th Edition was chosen to provide an estimate of intellectual ability because it provides a full-scale intelligence quotient and is able to assess the full age range of our study sample.

#### Outcome assessments

Treatment outcome measures were selected to elucidate a comprehensive set of behaviors. This included measures of global behavioral disturbance, social function, repetitive behaviors, and anxiety. The Aberrant Behavior Checklist (ABC) community version [46] and the CGI-improvement (CGI-I)-global were used to assess global behavioral disturbance. The ABC was designed to objectively identify five behavior subscales through observation by the primary caregiver. Items are rated on a four-point Likert scale, with higher scores indicating more severe problems. We used three subscales: irritability, hyperactivity, and social withdrawal. The CGI-I global is a well validated clinician-rated measure that employs a seven point scale (1 = very much improved to 7 = very much worse) to determine the patient’s response to treatment. The Social Responsiveness Scale (SRS) [47] and the Clinical Global Impressions Improvement Scale (CGI-I)-Social [43] were used to measure social function. The SRS is a 65-item caregiver rating scale of social behaviors specific to ASD and provides a total score that quantifies ASD severity. The CGI-I social allows clinicians to provide an impression of improvement (1 = very much improved to 7 = very much worse) on a seven-point Likert Scale. The Child Yale-Brown Obsessive Compulsive Scale (CY-BOCS; [44], modified by [45]) and the Repetitive Behavior Scale-Revised (RBS-R) [48] were used to measure repetitive behaviors. The CY-BOCS is a clinician-rated questionnaire measuring the time spent, distress, interference, resistance, and control in relation to obsessions and compulsions based on a five-point scale. The RBS-R was developed to capture the breadth of repetitive behaviors specific to autism and is a 43-item parent report measure. Lastly, the Behavioral Assessment System for Children 2nd Edition (BASC-2) [49] anxiety scale and the Child and Adolescent Symptom Inventory (CASI) [50] anxiety component were used to measure symptoms of anxiety. These outcome measures were completed at the baseline visit, week 8 and week 16. Research bloodwork included cytokines (IL-1β IL-6, IL-10, and TNF-α) and was performed at screening and week 16. Cytokine levels, including IL1-β, IL-10, and TNF-α in plasma; IL-6 in serum, were measured by ELISA using standard manufacturer’s protocol. ELISA kits were selected based on range of sensitivity by manufacturer and previous reports. Samples were run in duplicate using human TNF-α quantikine HS ELISA (R&D systems), human IL-6 quantikine HS ELISA (R&D systems), human IL1-β ELISA (Ray Biotech), and human IL-10 ELISA (Ray Biotech). Cytokine levels (pg/ml) were detectable in all IL-6 samples and all but one TNF-α and IL1-β samples, and all but two IL-10 samples. Specifically, one baseline sample was below detectable levels for TNF-α, IL10, and IL1-β and one baseline sample was below detectable for IL-10. These samples were treated as missing values and not included in the analysis.

#### Safety assessments

Safety was assessed using the Clinical Global Impressions Improvement Scale (CGI-I)-Global and the Safety Monitoring Uniform Report Form (SMURF). If a participant had scored 6 or greater for two or more weeks, they would have been discontinued from the study. At each study visit, the treating physician completed the SMURF [51], assessed vital signs, height, and weight. Funduscopic exam was performed at weeks 8 and 16. Clinical laboratory tests for safety included bloodwork [complete blood count (CBC), electrolytes, blood urea nitrogen (BUN), creatinine, alanine aminotransferase (ALT), aspartate aminotransferase (AST), insulin, fasting glucose, and creatine kinase (CK)] and electrocardiogram performed at screening and weeks 4, 8, and 16. Urinalysis for micro- and macroscopic hematuria was performed at screening and week 16. A urine pregnancy test was performed at screening in any menstruating girl.

### Statistical analysis

All analyses compared baseline visit to the week 16 visit, and controlled for participant’s age. One subject demonstrated a placebo response and was removed from the analyses. We used repeated measures ANOVA to model the outcomes at each of the study visits, with a planned contrast statement to assess the change from baseline to week 16. CGI scores at weeks 8 and 16 were classified as responders if they scored 1 or 2 on the measure. The percentage of responders is presented with exact 95% confidence limits.

Change in cytokine levels (pg/ml) from baseline to week 16 was highly skewed and, therefore, was assessed using the Wilcoxon signed rank test. We used Spearman correlation coefficients to examine the degree of association between baseline cytokine levels and their change from baseline to the end of the study with the change in outcome measures. Only cytokines and outcomes that showed a significant difference over the course of the study were included in this analysis.

## Results

### Participant attrition and baseline characteristics

**Fig. 1:**
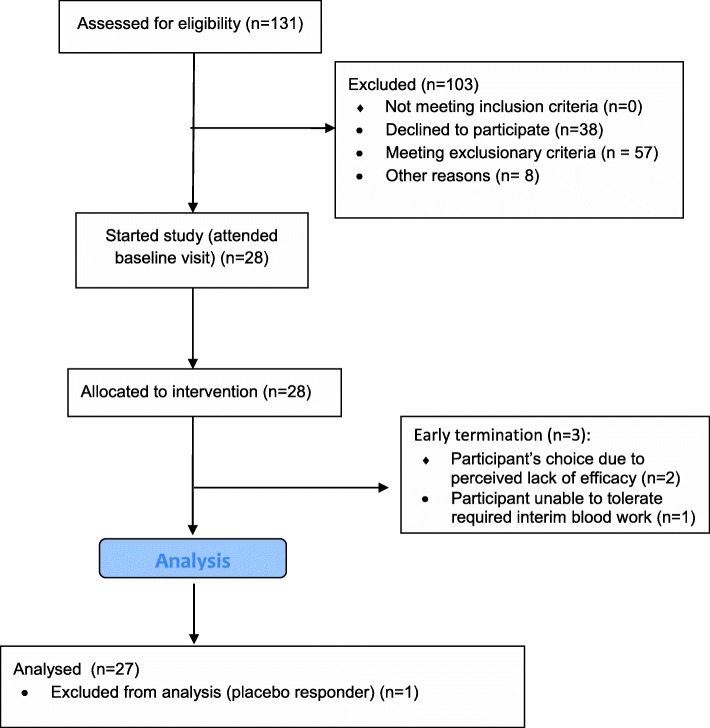
CONSORT 2010 flow diagram

Potential participants were screened over the phone (131). Twenty-eight participants started treatment and 25 participants completed the study (see Fig. 1). Baseline characteristics are summarized in Table 1. Refer to Additional file 1: Table S1 for a list of educational and dietary interventions being received by participants.

**Table 1:** Sample characteristics

Age in years [mean (SD)]	6.3 (2.4)
Male:Female	13:1
IQ [Mean (SD)]	69.9 (26.5)
ADOS score [mean (SD)]	17.1 (5.7)
Concomitant IBI (%)	10.7
Concomitant ABA (%)	21.4
Concomitant social skills group (%)	21.4
Diagnosis	# patients with comorbidity (/28)
Anxiety NYD	4
Specific phobia	2
ADHD	3
OCD	1

*Abbreviations: IBI* intensive behavioral therapy, *ABA* applied behavior analysis, *NYD* not yet diagnosed, *ADHD* attention deficit/hyperactivity disorder, *OCD* obsessive compulsive disorder

### Maximum tolerated dose and overall safety

There were no serious adverse events (SAEs) in any of the doses within the range tested (0.25 mg/kg, 0.5 mg/kg, and 0.75 mg/kg) and as such the maximum tolerated dose was determined to be 0.75 mg/kg once daily.

Overall, pioglitazone was well tolerated (see Table 2). Two participants discontinued intervention due to perceived non-efficacy and one due to the inability to tolerate interim blood work during the study. Adverse events (AEs) were of mild to moderate severity. Thirty-nine percent of the sample experienced upper respiratory tract infection (URTI). Three participants experienced mild neutropenia [see Additional file 1: Table S2 (a–c) for complete blood count results for each of these participants]. In one participant this was a pre-existing condition, in another it resolved while staying on medication, and in the third it only presented during the final visit and spontaneously recovered. As such, it was deemed to be unlikely related to the study intervention. BMI did not change significantly during the study [mean change 0.12; 95% confidence interval (− 0.03; 0.28) *p* = 0.1].

**Table 2:** Adverse events

Event	# Participants
Eye disorders	
Eye redness	1
Oral/gastrointestinal disorders	
Vomiting	3
Abdominal pain	2
Constipation	1
Diarrhea	1
Mouth sore/dry lips	2
Tooth problem	6
General disorders	
Increased energy/behavioral activation	3
Fatigue	1
Increased sweating	1
Immune system disorders	
Neutropenia	3
Infections	
Fever	1
Gastroenteritis	3
Upper respiratory tract infection	11
Injury	
Skin bruise	1
Skin burn	1
Insect bite	2
Appetite	
Decreased appetite	2
Increased appetite	1
Musculoskeletal	
Muscle pain/cramps	2
Nervous system disorder	
Concentration difficulty	1
Headache	1
Psychiatric disorders	
Disinhibition/silliness	2
Increased impulsivity	1
Emotional lability	2
Increased aggression/irritation/anger	5
Insomnia (initial/mid-cycle)	6
Negative thoughts	1
Self-injurious behavior	2
Genitourinary	
Diurnal/nocturnal enuresis	3
Increased urinary frequency	1
Genital discomfort	1
Respiratory	
Cough	1
Nasal congestion	2
Sleep apnea	1
Throat clearing	1
Skin	
Skin rash	2
Skin scratching/picking	3

### Preliminary evidence of efficacy

**Fig. 2:**
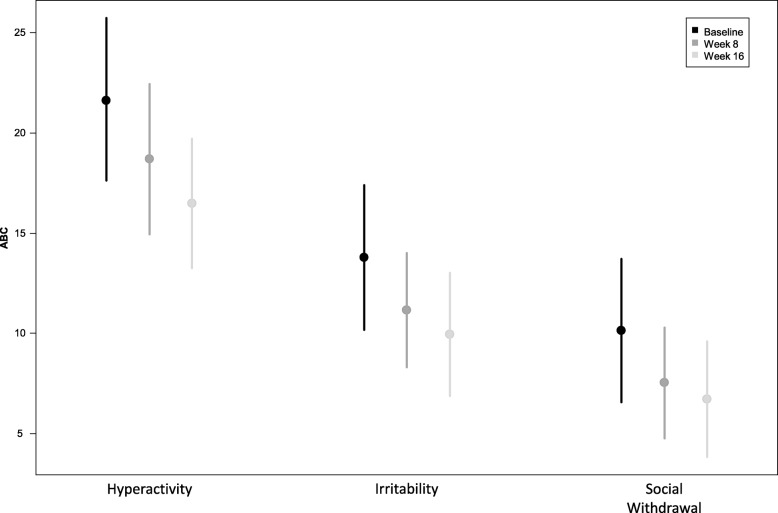
Change in ABC subscales with treatment

**Fig. 3:**
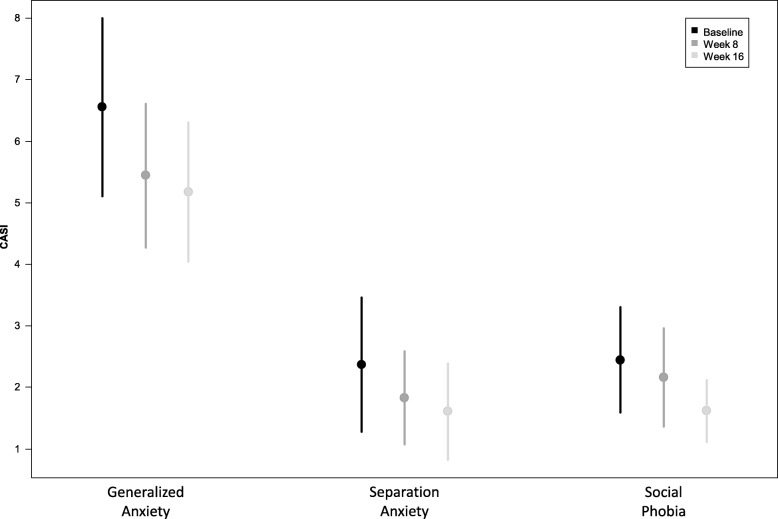
Change in anxiety with treatment

**Fig. 4:**
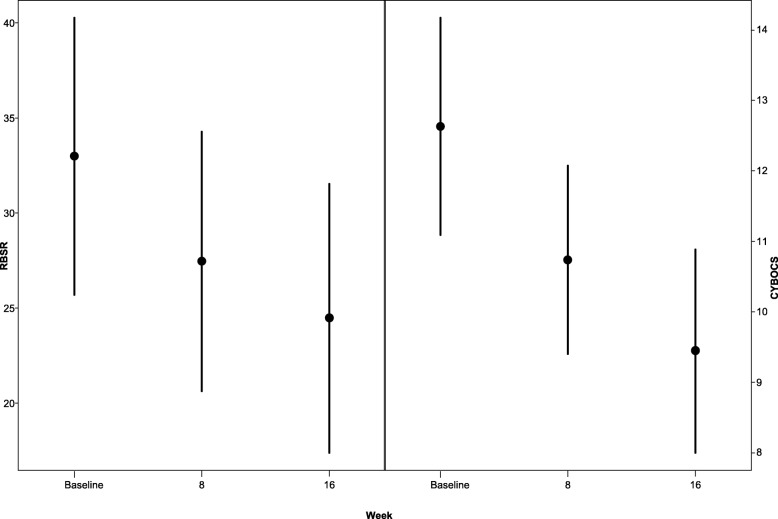
Change in repetitive behavior with treatment

One participant had a response to placebo at week 2 and was removed from the statistical analyses. Results from the remaining participants showed statistically significant changes in scores on global function, social function, irritability, hyperactivity, repetitive behaviors, and anxiety, although not on all measures (see Table 3, Additional file 1: Table S3, Figs. 2, 3, and 4). The outcome measures that remained statistically significant after using Bonferroni adjusted level for significance (*p* = 0.05/10 = *p* ≤ 0.005) were ABC social withdrawal, irritability and hyperactivity, as well as CYBOCS and RBS-R total score. Effect size calculated using the pooled standard deviation ranged from medium to large (0.43 to 0.9) on these measures (see Table 3).

**Table 3:** Change in outcome measures with treatment

	Estimate (95%CL)			Estimate (95%CL) *p* value	Effect size^d^ pooled sd
	Baseline	Week 8	Week 16	Week 16–baseline	
*n*	27	25	25		
Social function					
ABC social withdrawal	10.1 (6.6; 13.7)	7.5 (4.8; 10.3)	6.7 (3.8; 9.6)	− 3.4 (− 5.3; 1.6) 0.0005*	− 0.43 7.96
SRS total^a^	88.4 (73.6; 103.2)	88.2 (78.9; 97.5)	85.8 (75.2; 96.4)	− 2.6 (− 17.2; 12.0) 0.7	− 0.09 29.29
Externalizing behaviors					
ABC irritability	13.8 (10.2; 17.4)	11.2 (8.3; 14.0)	10.0 (6.9; 13.0)	− 3.8 (− 6.3; − 1.3) 0.004*	− 0.46 8.27
ABC hyperactivity	21.7 (17.6; 25.7)	18.7 (14.9; 22.4)	16.5 (13.2; 19.7)	− 5.2 (− 7.8; − 2.6) 0.0003*	− 0.58 8.96
Anxiety					
BASC-2 anxiety^b^	8.9 (5.5; 12.2)	8.1 (5.3; 10.8)	8.2 (5.2; 11.3)	− 0.6 (− 2.8; 1.5) 0.6	− 0.08 7.76
CASI-4 generalized anxiety^c^	6.6 (5.1; 8.0)	5.4 (4.3; 6.6)	5.2 (4.0; 6.3)	− 1.4 (− 2.5; − 0.3) 0.02	− 0.44 3.16
CASI-4 social phobia^c^	2.4 (1.6; 3.3)	2.2 (1.4; 3.0)	1.6 (1.1; 2.1)	− 0.8 (− 1.5; − 0.2) 0.01	− 0.50 1.66
CASI-4 separation anxiety^c^	2.4 (1.3; 3.5)	1.8 (1.1; 2.6)	1.6 (0.8; 2.4)	− 0.8 (− 1.6; 0.1) 0.08	− 0.33 2.32
Repetitive behaviors					
RBSR total	33.0 (25.7; 40.3)	27.5 (20.6; 34.3)	24.5 (17.4; 31.5)	− 8.5 (− 13.3; − 3.7) 0.0009^*^	− 0.48 17.62
CY-BOCS^a^	12.6 (11.1; 14.2)	10.7 (9.4; 12.1)	9.4 (8.0; 10.9)	− 3.2 (− 4.4; − 2.0) < .0001*	− 0.90 3.54
Clinical global impression	% Responders: score of 1 or 2 (95%CL)				
	Week 8			Week 16	
*n*	25			27	
CGI social	20.3 (9.4; 38.2)			34.1 (18.6; 54.0)	
CGI global	26.0 (11.7; 48.1)			45.8 (27.8; 65.0)	

Score (95%CL) at each visit is estimated from a repeated measures linear model controlling for age; a planned contrast is used to estimate the change from baseline to week 16 from the model; model excludes one participant with a placebo response at week 2

^a^*n* = 26 at baseline and week 16

^b^*n* = 24 at week 8

^c^*n* = 26 at week 16; * = remains significant using Bonferroni adjusted level for significance of *p* = 0.005

^d^Week 16–baseline/pooled standard deviation where the pooled standard deviation is derived from the baseline and week 16 standard deviations obtained from the repeated measures model

### Cytokine levels

**Fig. 5:**
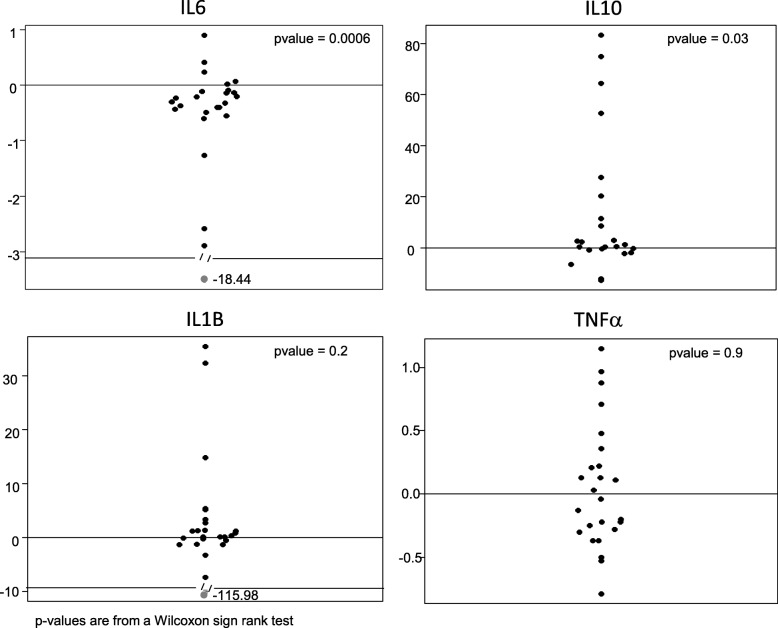
Change in cytokine value with treatment

Cytokine levels in serum (IL-6) and plasma (TNF-α, IL-10, IL1-β) were measured at screening and week 16 visits. Two participants were removed from these analyses. The first was the placebo responder noted in the last section, and the second was an outlier in that cytokine levels were significantly higher than the rest of the cohort. One participant provided the second sample at week 8 due to early withdrawal from the study, and it was analyzed as a week 16 per a last observation carried forward approach in keeping with the intent-to-treat principle. Figure 5 shows the change in each cytokine value with pioglitazone treatment. Additional file 2: Figure S3 shows cytokine concentrations as measured pre and post-treatment with pioglitazone. Significant changes with treatment occurred with both IL-6 and IL-10 (mean change (Wilcoxon sign rank *p* value) IL6: − 1.6 (*p* = 0.0006); IL-10: 13.6 (*p* = 0.03)) (Table 4). IL-1β and TNF-α did not change significantly with treatment. We then looked at the association between the two cytokines showing significant change with continuous outcome measures that also showed a significant change with treatment: ABC-social withdrawal, irritability, and hyperactivity, CY-BOCS, and CASI-generalized anxiety and social phobia. Baseline IL-6 levels were significantly positively correlated (0.5, *p* = 0.01) with the change in CYBOCS scores over the course of the study, but change in IL-6 levels did not correlate with change for any of the outcome measures. Baseline IL-10 levels were not significantly correlated with the change in any of the outcome measures. Change in IL-10 levels with treatment was significantly positively correlated with change in ABC social withdrawal scores (0.44, *p* = 0.05) and showed a trend (0.41, *p* = 0.06) toward a positive correlation with change in CASI-social phobia score.

**Table 4:** Cytokine levels measured at baseline and post-treatment

	Baseline		Post-treatment (*n* = 26)		*p* value
	Mean	s.d.	Mean	s.d.	
IL6 (pg/ml)	1.37	1.29	0.760	0.43	0.0006
IL1β (pg/ml)	13.5	34.8	14.4	20.3	> 0.05
TNF-α (pg/ml)	0.69	0.37	0.782	0.49	> 0.05
IL10 (pg/ml)	31.2	53.9	42.8	72.5	0.03

## Discussion

This study had three main objectives: Firstly, to identify the maximum tolerated dose of pioglitazone in autistic children within the range approved by Health Canada and the FDA. Secondly, to evaluate which of a number of outcome measures could best capture the therapeutic effect of pioglitazone in this population. Finally, to evaluate whether a biomarker, cytokine, levels in a peripheral blood sample significantly predict the effect of pioglitazone on outcome measures. The maximum tolerated dose of pioglitazone identified was 0.75 mg/kg in autistic children. No serious adverse events occurred at any of the three doses tested. None of the three participants with early termination dropped out due to adverse effects related to study drug, but rather related to perceived non-efficacy and an inability to tolerate study safety bloodwork. The most recent monograph of pioglitazone lists heart failure, hepatic insufficiency, and allergic reactions as significant side effects of pioglitazone. None of these side effects were observed in our study nor were they observed in either of the two previously published studies of pioglitazone in autistic children [36, 37]. Boris et al. [36] observed two incidents of slightly elevated white blood cell and glucose levels and three incidents of slightly elevated liver enzymes (ALT and AST), all resolved without intervention. Ghaleiha et al. [37] found that the most common adverse events were headache and vomiting, but the incidence was not statistically different between the pioglitazone group and the control group. A few studies have examined the safety and efficacy of pioglitazone and the related drug rosiglitazone (also a thiazolidinedione) in children and adolescents with diabetes and obesity and similarly found these drugs to be well tolerated [52–54]. Reported adverse events included transient elevation of liver enzymes, weight gain, edema, and hypoglycemia (only in rosiglitazone combined with metformin [52]). None of our participants experienced these adverse events. Three participants in our study experienced mild neutropenia, which was determined to be unlikely related given the time frame and course. Two cases of reversible neutropenia have been reported in adults with diabetes treated with pioglitazone [55, 56] and a retrospective study of 50 diabetic adults treated with pioglitazone showed a significant reduction in hemoglobin, hematocrit, platelets, and white blood cell count, compared to placebo suggesting a possible mild bone marrow suppressive effect of the drug [57]. Therefore, future studies should carefully monitor complete blood counts of patients taking pioglitazone. Thirty-nine percent of our participants experienced upper respiratory tract infection (URTI). URTI occurs very commonly in children, and because we did not have a placebo-arm for comparison, it was unclear if pioglitazone further predisposes children to URTI. URTI is noted in the product monograph for pioglitazone as a common occurrence (occurring in > 1% to < 10% of adult patients). Placebo-controlled trials of pioglitazone in adolescents did not list URTI as a common adverse event [52–54]. Future placebo-controlled trials in children should monitor closely for a significant risk of URTI.

We used a number of outcome measures to evaluate the potential efficacy of pioglitazone in autistic children. Consistent with the prior two studies of pioglitazone in ASD, we found significant improvement in irritability, hyperactivity, and social withdrawal within the dose range approved by regulatory agencies, as measured by the ABC. In addition, we identified significant improvement in repetitive behaviors and anxiety as measured by the CY-BOCS, RBS-R, and CASI-4 anxiety scales. The ABC social withdrawal, irritability, and hyperactivity as well as CY-BOCS and RBS-R total score remained significant after correction for multiple comparisons. Improvement in repetitive behavior as measured by the CY-BOCS had a large effect size, while changes in the ABC social withdrawal, irritability, hyperactivity, and RBS-R (for repetitive behavior) had a medium effect size. Using the standard deviations of scores on the ABC social withdrawal, irritability and hyperactivity subscales taken from Brown et al. [58], and the standard deviations of the total scores of the RBS-R and CYBOCS taken from Lam and Aman [48] and Scahill et al. [59], respectively, we found that the ABC social withdrawal, irritability, and hyperactivity subscale scores as well as RBS-R total score were just shy of − 0.5 SD (with mean change of − 0.42 to − 0.49 SDs). The CYPBOCS showed a mean change of − 1.14 SD. This along with effect sizes calculated from our data suggest that pioglitazone has the largest signal for reduction of repetitive behavior as measured by the CYBOCS. Using the CGI-global and social, clinicians rated 46% of participants as significantly improved in global functioning and 34% significantly improved in social functioning. These findings require confirmation in a larger, randomized, double-blind, placebo-controlled study, but do provide preliminary evidence that pioglitazone may improve both core ASD symptoms and frequently associated symptoms.

The direction of change in cytokines in our study (decreasing IL-6 and increasing IL-10) is consistent with the known effect of PPAR-gamma agonists like pioglitazone [60]. It was expected that IL-1β and TNF-α would also decrease with treatment. This was not observed in our study and may relate to the small sample size. One study did not find that thiazolidinediones were able to suppress TNF-α and IL-6 in macrophages [61]. While baseline IL-6 levels were correlated with worse CYBOCS scores as expected, reduction of IL-6 with treatment was not correlated with outcome measures. Change in IL-10 with treatment was correlated with outcome measures but contrary to our expectations. This was contrary to expectation given studies such as one examining children with 22q11 deletion syndrome (at high risk of autism) that found that level of IL-1β in the blood, and the ratio of IL-6:IL10 in serum was significantly associated with social scores on the ADI-R [62]. This again may relate to the small sample size in our study. Alternatively, peripheral cytokine levels do not directly reflect cytokine levels in the CNS, nor do they reflect immune activation and inflammation in the CNS [4]. Pioglitazone is able to cross the blood-brain barrier and PPAR-ϒ is expressed in the following areas of CNS: mostly microglia and astrocytes, less so in hippocampal dentate gyrus, basal ganglia, thalamus, and piriform cortex [63–65]. Pioglitazone is able to inhibit the secretion of TNF-α, IL-1β, and IL-6 and enhance IL-10 secretion in mouse astrocytes [66]. Moreover, pioglitazone can affect mitochondrial biogenesis and reduce currents through the NMDA glutamate receptor, two alternative mechanisms that may be important in autism [31–35]. Such alternative mechanisms could explain the improvement seen on outcome measures during treatment with pioglitazone in our study, but without a direct correlate with peripheral cytokine levels. Given that IL-6 and IL-10 both changed as expected with pioglitazone treatment, it may be worth repeating these analyses in a larger study.

## Limitations

This study had some important limitations to consider. Firstly, this study was primarily designed as a pilot maximum tolerated dose (MTD) study. The study used a single arm with no placebo control group with which to compare change in behavioral outcome measures and cytokine levels. Additionally, the sample size may be too small to detect cytokine change with treatment and to find an association with behavior outcome measures. A typically developing control group may help identify a subgroup of autistic children having abnormal cytokine levels and may more accurately demonstrate a relationship with outcome measures.

In summary, we identified 0.75 mg/kg of pioglitazone to be the maximum tolerated dose within the range allowed by Health Canada and the FDA using a MTD design. Pioglitazone was well tolerated with no serious adverse events identified. Pioglitazone demonstrated efficacy in improving both core (social withdrawal and repetitive behaviors) and associated (irritability, hyperactivity, and anxiety) autism symptoms and did significantly decrease the pro-inflammatory cytokine IL-6 and increase the anti-inflammatory cytokine IL-10. These findings indicate the need for a larger randomized controlled trial with potential emphasis on pioglitazone’s efficacy in reducing repetitive behavior as measured by the CYBOCS, to replicate these promising findings.

## Additional files

Additional file 1: **Table S1.** Concurrent educational and dietary interventions received by participants. **Table S2a.** Complete blood count and corresponding visit for participant 1 with mild neutropenia. **Table S2b.** Complete blood count and corresponding visit for participant 2 with mild neutropenia. **Table S2c.** Complete blood count and corresponding visit for participant 3 with mild neutropenia. **Table S3.** Change from baseline to week 16 by dose. (DOCX 162 kb)

Additional file 2: Cytokine concentrations pre and post-treatment with pioglitazone. (PDF 44 kb)

## Abbreviations

ABC: Aberrant Behavior Checklist
ADI-R: Autism Diagnostic Interview-Revised
ADOS: Autism Diagnostic Observational Schedule
ASD: Autism spectrum disorder
BASC-2: Behavioral Assessment System for Children 2nd Edition
BLC: B-lymphocyte chemoattractant
CASI: Child and Adolescent Symptom Inventory
CGI-I: Clinician Global Impression scale-Improvement
CGI-S: Clinician Global Impression scale-Severity
Ckβ8.1: Chemokine-β8
CNS: Central nervous system
CSF: Cerebrospinal fluid
CY-BOCS: Child Yale-Brown Obsessive Compulsive Scale
DSM-5: Diagnostic and Statistical Manual of Mental Disorders 5th edition
DSM-IV-TR: Diagnostic and Statistical Manual of Mental Disorders 4th edition, text revision
ELIZA: Enzyme-linked immunosorbent assay
GCP: Good clinical practice
GFAP: Glial fibrillary acidic protein
GM-CSF: Granulocyte-macrophage colony stimulating factory
ICH: International Council for Harmonization
IFN-ϒ: Interferon-ϒ
IL: Interleukin
MCP: Macrophage chemoattractant protein
MDC: Macrophage-derived chemokine
MIG: Monokine induced by interferon-ϒ
MTD: Maximum tolerated dose
NAP-2: Neutrophil activating peptide-2
NMDA: N-methyl-d-aspartate
PET: Positron emission tomography
PPAR-ϒ: Peroxisome proliferator activated receptor - gamma
RBS-R: Repetitive Behavior Scale-Revised
SAE: Serious adverse event
SMURF: Safety Monitoring Uniform Report Form
SRS: Social Responsiveness Scale
TARC: Thymus activation-regulated chemokine
TGF-1β: Transforming growth factor-β1
TNF-α: Tumor necrosis factor-α
TZDs: Thiazolidinediones
URTI: Upper respiratory tract infection

## Footnotes

1 We acknowledge that there is no consensus within the community about whether to use identity-first (i.e. autistic individual) language, or person-first language (i.e. person with autism). We are choosing to use the term autistic individual in this paper, but respect both perspectives.

## References

[1] Association AP (2013). Diagnostic and statistical manual of mental disorders (DSM-5®).

[2] Baio J, Wiggins L, Christensen DL, Maenner MJ, Daniels J, Warren Z, Kurzius-Spencer M, Zahorodny W, Robinson Rosenberg C, White T (2018). Prevalence of autism spectrum disorder among children aged 8 years—autism and developmental disabilities monitoring network, 11 Sites, United States, 2014. MMWR Surveill Summ.

[3] Nadeem A, Ahmad SF, Attia SM, Bakheet SA, Al-Harbi NO, Al-Ayadhi LY (2018). Activation of IL-17 receptor leads to increased oxidative inflammation in peripheral monocytes of autistic children. Brain Behav Immun.

[4] Pardo CA, Farmer CA, Thurm A, Shebl FM, Ilieva J, Kalra S, Swedo S (2017). Serum and cerebrospinal fluid immune mediators in children with autistic disorder: a longitudinal study. Mol Autism.

[5] Jyonouchi H, Geng L, Streck DL, Dermody JJ, Toruner GA (2017). MicroRNA expression changes in association with changes in interleukin-1ss/interleukin10 ratios produced by monocytes in autism spectrum disorders: their association with neuropsychiatric symptoms and comorbid conditions (observational study). J Neuroinflammation.

[6] Ahmad SF, Nadeem A, Ansari MA, Bakheet SA, Al-Ayadhi LY, Attia SM (2017). Upregulation of IL-9 and JAK-STAT signaling pathway in children with autism. Prog Neuro-Psychopharmacol Biol Psychiatry.

[7] Al-Ayadhi LY, Mostafa GA (2012). Elevated serum levels of interleukin-17A in children with autism. J Neuroinflammation.

[8] Garay PA, McAllister AK (2010). Novel roles for immune molecules in neural development: implications for neurodevelopmental disorders. Front Synaptic Neurosci.

[9] Ashwood P, Wakefield AJ (2006). Immune activation of peripheral blood and mucosal CD3+ lymphocyte cytokine profiles in children with autism and gastrointestinal symptoms. J Neuroimmunol.

[10] Prata J, Santos SG, Almeida MI, Coelho R, Barbosa MA (2017). Bridging autism spectrum disorders and schizophrenia through inflammation and biomarkers—pre-clinical and clinical investigations. J Neuroinflammation.

[11] Pardo CA, Vargas DL, Zimmerman AW (2005). Immunity, neuroglia and neuroinflammation in autism. Int Rev Psychiatry.

[12] Vargas DL, Nascimbene C, Krishnan C, Zimmerman AW, Pardo CA (2005). Neuroglial activation and neuroinflammation in the brain of patients with autism. Ann Neurol.

[13] Zimmerman AW, Jyonouchi H, Comi AM, Connors SL, Milstien S, Varsou A, Heyes MP (2005). Cerebrospinal fluid and serum markers of inflammation in autism. Pediatr Neurol.

[14] Suzuki K, Sugihara G, Ouchi Y, Nakamura K, Futatsubashi M, Takebayashi K, Yoshihara Y, Omata K, Matsumoto K, Tsuchiya KJ (2013). Microglial activation in young adults with autism spectrum disorder. JAMA Psychiatry.

[15] Rodriguez JI, Kern JK (2011). Evidence of microglial activation in autism and its possible role in brain underconnectivity. Neuron Glia Biol.

[16] Ashwood P, Enstrom A, Krakowiak P, Hertz-Picciotto I, Hansen RL, Croen LA, Ozonoff S, Pessah IN, Van de Water J (2008). Decreased transforming growth factor beta1 in autism: a potential link between immune dysregulation and impairment in clinical behavioral outcomes. J Neuroimmunol.

[17] Ashwood P, Anthony A, Torrente F, Wakefield AJ (2004). Spontaneous mucosal lymphocyte cytokine profiles in children with autism and gastrointestinal symptoms: mucosal immune activation and reduced counter regulatory interleukin-10. J Clin Immunol.

[18] D'Mello C, Swain MG (2017). Immune-to-brain communication pathways in inflammation-associated sickness and depression. Curr Top Behav Neurosci.

[19] McCusker RH, Kelley KW (2013). Immune-neural connections: how the immune system's response to infectious agents influences behavior. J Exp Biol.

[20] Masi A, Quintana DS, Glozier N, Lloyd AR, Hickie IB, Guastella AJ (2015). Cytokine aberrations in autism spectrum disorder: a systematic review and meta-analysis. Mol Psychiatry.

[21] Masi A, Glozier N, Dale R, Guastella AJ (2017). The immune system, cytokines, and biomarkers in autism spectrum disorder. Neurosci Bull.

[22] Goines P, Van de Water J (2010). The immune system's role in the biology of autism. Curr Opin Neurol.

[23] Bryn V, Aass HC, Skjeldal OH, Isaksen J, Saugstad OD, Ormstad H (2017). Cytokine profile in autism spectrum disorders in children. J Mol Neurosci.

[24] Ashwood P, Krakowiak P, Hertz-Picciotto I, Hansen R, Pessah I, Van de Water J (2011). Elevated plasma cytokines in autism spectrum disorders provide evidence of immune dysfunction and are associated with impaired behavioral outcome. Brain Behav Immun.

[25] Tsilioni I, Taliou A, Francis K, Theoharides TC (2015). Children with autism spectrum disorders, who improved with a luteolin-containing dietary formulation, show reduced serum levels of TNF and IL-6. Transl Psychiatry.

[26] Hashim H, Abdelrahman H, Mohammed D, Karam R (2013). Association between plasma levels of transforming growth factor-β1, IL-23 and IL-17 and the severity of autism in Egyptian children. Res Autism Spectr Disord.

[27] Landreth GE, Heneka MT (2001). Anti-inflammatory actions of peroxisome proliferator-activated receptor gamma agonists in Alzheimer's disease. Neurobiol Aging.

[28] Liu HR, Tao L, Gao E, Lopez BL, Christopher TA, Willette RN, Ohlstein EH, Yue TL, Ma XL (2004). Anti-apoptotic effects of rosiglitazone in hypercholesterolemic rabbits subjected to myocardial ischemia and reperfusion. Cardiovasc Res.

[29] Schutz B, Reimann J, Dumitrescu-Ozimek L, Kappes-Horn K, Landreth GE, Schurmann B, Zimmer A, Heneka MT (2005). The oral antidiabetic pioglitazone protects from neurodegeneration and amyotrophic lateral sclerosis-like symptoms in superoxide dismutase-G93A transgenic mice. J Neurosci.

[30] Wu ZH, Zhao SP, Chu LX, Ye HJ (2010). Pioglitazone reduces tumor necrosis factor-alpha serum concentration and mRNA expression of adipose tissue in hypercholesterolemic rabbits. Int J Cardiol.

[31] Almasi-Nasrabadi M, Javadi-Paydar M, Mahdavian S, Babaei R, Sharifian M, Norouzi A, Dehpour AR (2012). Involvement of NMDA receptors in the beneficial effects of pioglitazone on scopolamine-induced memory impairment in mice. Behav Brain Res.

[32] Ghosh S, Patel N, Rahn D, McAllister J, Sadeghi S, Horwitz G, Berry D, Wang KX, Swerdlow RH (2007). The thiazolidinedione pioglitazone alters mitochondrial function in human neuron-like cells. Mol Pharmacol.

[33] Siddiqui MF, Elwell C, Johnson MH (2016). Mitochondrial dysfunction in autism spectrum disorders. Autism Open Access.

[34] Lee EJ, Choi SY, Kim E (2015). NMDA receptor dysfunction in autism spectrum disorders. Curr Opin Pharmacol.

[35] Rossignol DA, Frye RE (2012). Mitochondrial dysfunction in autism spectrum disorders: a systematic review and meta-analysis. Mol Psychiatry.

[36] Boris M, Kaiser CC, Goldblatt A, Elice MW, Edelson SM, Adams JB, Feinstein DL (2007). Effect of pioglitazone treatment on behavioral symptoms in autistic children. J Neuroinflammation.

[37] Ghaleiha A, Rasa SM, Nikoo M, Farokhnia M, Mohammadi MR, Akhondzadeh S. A pilot double-blind placebo-controlled trial of pioglitazone as adjunctive treatment to risperidone: effects on aberrant behavior in children with autism. 2015;229(1-2):181–87. 10.1016/j.psychres.2015.07.043 26208985

[38] Association AP (2000). DSM-IV-TR: diagnostic and statistical manual of mental disorders, text revision.

[39] Lord C, Rutter M, Goode S, Heemsbergen J, Jordan H, Mawhood L, Schopler E (1989). Autism diagnostic observation schedule: a standardized observation of communicative and social behavior. J Autism Dev Disord.

[40] Gotham K, Risi S, Pickles A, Lord C (2007). The autism diagnostic observation schedule: revised algorithms for improved diagnostic validity. J Autism Dev Disord.

[41] Lord C, Rutter M, DiLavore PC, Risi S, Gotham K, Guthrie W, Bishop SL (2012). Autism diagnostic observation schedule, second edition (ADOS-2) manual (part I): modules 1 - 4.

[42] Lord C, Rutter M, Le Couteur A (1994). Autism diagnostic interview-revised: a revised version of a diagnostic interview for caregivers of individuals with possible pervasive developmental disorders. J Autism Dev Disord.

[43] Guy W (1976). ECDEU assessment manual for psychopharmacology, revised.

[44] Goodman WK, Price LH, Rasmussen SA, Mazure C, Fleischmann RL, Hill CL, Heninger GR, Charney DS (1989). The Yale-Brown obsessive compulsive scale: I. Development, use, and reliability. Arch Gen Psychiatry.

[45] Scahill L, Riddle MA, McSwiggin-Hardin M, Ort SI, King RA, Goodman WK, Cicchetti D, Leckman JF (1997). Children's Yale-Brown obsessive compulsive scale: reliability and validity. J Am Acad Child Adolesc Psychiatry.

[46] Aman MG, Singh NN, Stewart AW, Field CJ (1985). The aberrant behavior checklist: a behavior rating scale for the assessment of treatment effects. Am J Ment Defic.

[47] Constantino JN, Gruber CP (2012). Social responsiveness scale (SRS).

[48] Lam KS, Aman MG (2007). The repetitive behavior scale-revised: independent validation in individuals with autism spectrum disorders. J Autism Dev Disord.

[49] Reynolds CR, Kamphaus R (2006). BASC-2: behavior assessment system for children.

[50] Sukhodolsky DG, Scahill L, Gadow KD, Arnold LE, Aman MG, McDougle CJ, McCracken JT, Tierney E, Williams White S, Lecavalier L (2008). Parent-rated anxiety symptoms in children with pervasive developmental disorders: frequency and association with core autism symptoms and cognitive functioning. J Abnorm Child Psychol.

[51] Greenhill LL, Vitiello B, Fisher P, Levine J, Davies M, Abikoff H, Chrisman AK, Chuang S, Findling RL, March J (2004). Comparison of increasingly detailed elicitation methods for the assessment of adverse events in pediatric psychopharmacology. J Am Acad Child Adolesc Psychiatry.

[52] Zeitler P, Hirst K, Pyle L, Linder B, Copeland K, Arslanian S, Cuttler L, Nathan DM, Tollefsen S, Group TS (2012). A clinical trial to maintain glycemic control in youth with type 2 diabetes. N Engl J Med.

[53] Tafuri KS, Godil MA, Lane AH, Wilson TA (2013). Effect of pioglitazone on the course of new-onset type 1 diabetes mellitus. J Clin Res Pediatr Endocrinol.

[54] Zdravkovic V, Hamilton JK, Daneman D, Cummings EA (2006). Pioglitazone as adjunctive therapy in adolescents with type 1 diabetes. J Pediatr.

[55] Digman C, Klein AK, Pittas AG (2005). Leukopenia and thrombocytopenia caused by thiazolidinediones. Ann Intern Med.

[56] Karakurt F, Kargili A, Kasapoglu B (2010). Pioglitazone induced reversible pancytopenia. Exp Clin Endocrinol Diabetes.

[57] Berria R, Glass L, Mahankali A, Miyazaki Y, Monroy A, De Filippis E, Cusi K, Cersosimo E, Defronzo RA, Gastaldelli A (2007). Reduction in hematocrit and hemoglobin following pioglitazone treatment is not hemodilutional in type II diabetes mellitus. Clin Pharmacol Ther.

[58] Brown EC, Aman MG, Havercamp SM (2002). Factor analysis and norms for parent ratings on the aberrant behavior checklist-community for young people in special education. Res Dev Disabil.

[59] Scahill L, Sukhodolsky DG, Anderberg E, Dimitropoulos A, Dziura J, Aman MG, McCracken J, Tierney E, Hallett V, Katz K (2016). Sensitivity of the modified Children's Yale-Brown obsessive compulsive scale to detect change: results from two multi-site trials. Autism.

[60] Jiang C, Ting AT, Seed B (1998). PPAR-gamma agonists inhibit production of monocyte inflammatory cytokines. Nature.

[61] Thieringer R, Fenyk-Melody JE, Le Grand CB, Shelton BA, Detmers PA, Somers EP, Carbin L, Moller DE, Wright SD, Berger J (2000). Activation of peroxisome proliferator-activated receptor gamma does not inhibit IL-6 or TNF-alpha responses of macrophages to lipopolysaccharide in vitro or in vivo. J Immunol.

[62] Ross HE, Guo Y, Coleman K, Ousley O, Miller AH (2013). Association of IL-12p70 and IL-6:IL-10 ratio with autism-related behaviors in 22q11.2 deletion syndrome: a preliminary report. Brain Behav Immun.

[63] Braissant O, Foufelle F, Scotto C, Dauca M, Wahli W (1996). Differential expression of peroxisome proliferator-activated receptors (PPARs): tissue distribution of PPAR-alpha, -beta, and -gamma in the adult rat. Endocrinology.

[64] Moreno S, Farioli-Vecchioli S, Ceru MP (2004). Immunolocalization of peroxisome proliferator-activated receptors and retinoid X receptors in the adult rat CNS. Neuroscience.

[65] Bernardo A, Levi G, Minghetti L (2000). Role of the peroxisome proliferator-activated receptor-gamma (PPAR-gamma) and its natural ligand 15-deoxy-Delta12, 14-prostaglandin J2 in the regulation of microglial functions. Eur J Neurosci.

[66] Qiu D, Li XN (2015). Pioglitazone inhibits the secretion of proinflammatory cytokines and chemokines in astrocytes stimulated with lipopolysaccharide. Int J Clin Pharmacol Ther.

