# Supplementary material for: A pilot dose finding study of pioglitazone in autistic children
Source: Mol Autism. 2018 Nov 26;9:59. doi: 10.1186/s13229-018-0241-5 (PMC6258310; doi:10.1186/s13229-018-0241-5)
Supplement: Supplementary file 1 — Table S1. Concurrent educational and dietary interventions received by participants. Table S2a. Complete blood count and corresponding visit for participant 1 with mild neutropenia. Table S2b. Complete blood count and corresponding visit for participant 2 with mild neutropenia. Table S2c. Complete blood count and corresponding visit for participant 3 with mild neutropenia. Table S3. Change from baseline to week 16 by dose. (DOCX 162 kb) [file 13229_2018_241_MOESM1_ESM.docx]

Additional file 1

Table S1 Concurrent educational and dietary interventions received by participants

| *Intervention* | *# of participants* |
| --- | --- |
| Special diet | 2 |
| Supplements* | 57 |
| Educational intervention | 1 |
| Melatonin | 8 |
| Laxative | 2 |
| OT | 3 |
| SLP | 8 |
| Other ** | 3 |

* Supplement subtypes = vitamins (2), minerals (7), multivitamins (16), fatty acids (13), homeopathy/probiotics (13)

**Other: day camp, group therapy, music therapy

Table S2a. Complete blood count and corresponding visit for participant 1 with mild neutropenia

Table S2b. Complete blood count and corresponding visit for participant 2 with mild neutropenia

Table S2c. Complete blood count and corresponding visit for participant 3 with mild neutropenia

**Table S3**: Change from baseline to week 16 by dose

|  | Pioglitazone dose | | |
| --- | --- | --- | --- |
|  | 0.25 mg/Kg | 0.5 mg/Kg | 0.75 mg/Kg |
| **n** | 4 | 5 | 17 |
| **Social Function** |  |  |  |
| ABC Social Withdrawal^1^ | -4.8 (3.2) | -3.2 (5.0) | -2.9 (5.7) |
| SRS Total^1^ | -19.8 (19.4) | -4.2 (46.8) | 3.3 (43.8) |
| **Externalizing Behaviors** |  |  |  |
| ABC Irritability^1^ | -2.8 (2.9 | -2.2 (5.2) | -4.1 (6.0) |
| ABC Hyperactivity^1^ | -4.0 (2.2) | -6.8 (5.4) | -4.6 (6.4) |
| **Anxiety** |  |  |  |
| BASC-2 Anxiety | -3.3 (3.9) | 1.0 (6.7) | -0.5 (6.4) |
| CASI-4 Generalized Anxiety | -4.5 (2.6) | -2.2 (1.3) | -0.3 (2.0) |
| CASI-4 Social Phobia | -1.5 (1.0) | -1.2 (1.8) | -0.5 (1.7) |
| CASI-4 Separation Anxiety | -0.8 (3.3) | -0.6 (1.1) | -0.8 (2.4) |
| **Repetitive Behaviors** |  |  |  |
| RBSR Total^1^ | -5.8 (6.2) | -4.4 (4.5) | -2.2 (2.2) |
| CY-BOCS | 0.0 (0.2) | -0.1 (0.3) | 0.2 (0.5) |

^1^ dose 0.75 n=16
