# Supplementary figures and images for: A pilot dose finding study of pioglitazone in autistic children
Source: Mol Autism. 2018 Nov 26;9:59. doi: 10.1186/s13229-018-0241-5 (PMC6258310; doi:10.1186/s13229-018-0241-5)

**A**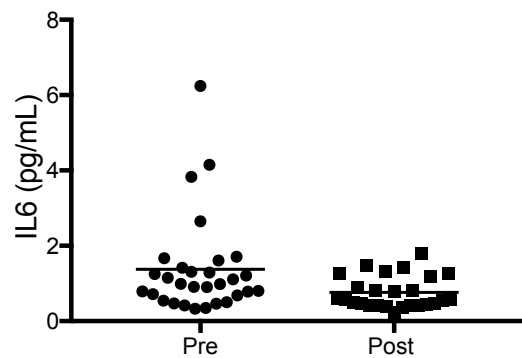**B**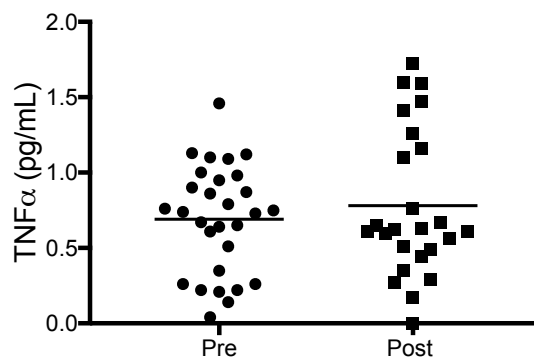**C**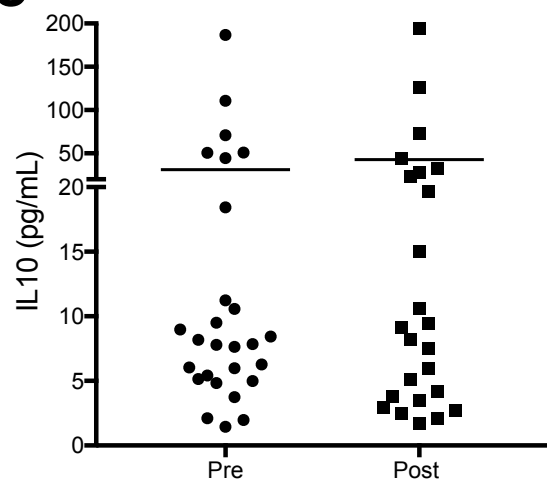**D**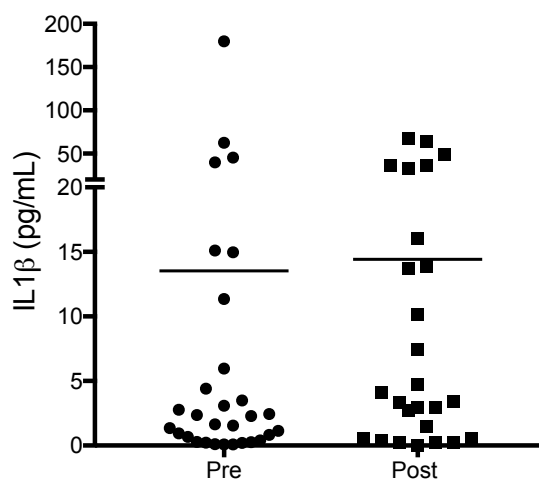

Supplement: Supplementary file 2 — Cytokine concentrations pre and post-treatment with pioglitazone. (PDF 44 kb) [file 13229_2018_241_MOESM2_ESM.pdf]
